# Materia Medica and Therapeutics

**Published:** 1878-08

**Authors:** 


### Materia Medica and Therapeutics.

The Explanation of Morphia Myosis.—Picard. (*Med. Press & Circ.*, May 22, '78.)

At a recent meeting of the Biological Society of Paris, Picard attributed the contraction of the pupil under the influence of morphia to paresis of the sympathetic ; for if the submaxillary gland is bared, and a small vein opened, the flow of blood is increased after an injection of the drug. Hence he concludes that it produces diminution of power of the great sympathetic.

Alcohol.—Carpenter. (*Br. Med. Journ.*, May 18, '78.)

For the subject of the annual oration before the Medical Society of London, Dr. Alfred Carpenter, the orator, selected the important topic of Alcoholic Drinks, in their various relations. We abstract the following points, relating principally to their physiological action.

In reply to the often asked question whether force can be produced by the ingestion of alcohol, after a careful review of both sides, he takes the affirmative, seeing no other explanation of the fact that many lives have been prolonged for months without other nourishment than that contained in spirituous liquors. He alluded to the fact that when dilute solutions of about the strength in which it would be found in the body, say 1 to 1,000, are passed through six inches of silicated carbon filter, the alcohol is metamorphosed into complex products, especially glycol, which should be considered one of the saccharine group. If this be a possible contingency, it may be transformed into force in the human economy under certain unknown circumstances, and as such be useful without danger as a luxury.

He inclined to the belief that the statements that alcohol acts upon the blood corpuscles and on the fibrin of the liquor sanguinis, were true. It is said that 1 part to 500 of blood interferes with the power of the blood to absorb oxygen (hence the purplish color of countenance of the bloated drinker) ; that it abstracts water from the blood-discs, and makes them adhere, and that it causes coagulation of the plastic elements of the blood, which thus collects in the capillaries, while the current may even thereby be stopped.

By such means the free administration of alcoholic stimulants to the weak or debilitated, especially after hæmorrhage, etc., has led to embolism ; or a clot would rapidly form in the heart, and the patient die. Such cases of embolism may get better, provided their alcohol be discontinued. Observation also shows that the elimination of carbonic dioxide is decreased after the imbibition of alcohol. These are undisputed facts with reference to large doses, but it does not follow that the weaker drinks, such as wine or beer, must cause such results.

It appears to be true that when once the system has been exposed to the full influence of alcohol, such as to cause results as above, then, but not till then, a cumulative action may begin ; an action which shall develop an instinctive desire for more.

According to its dilution, alcohol passes rapidly through membrane, acting upon it in its passage ; it is thus quickly conveyed to all parts of the system, and we cannot predicate where it will produce its first effect. Slight changes in the blood and tissues are not noticed, but when the altered blood corpuscles attain a certain percentage of the whole number, evil must result. Coagulated fibrin may be removed in small quantities, but it will soon gravitate, if not removed, into some organ, and impede circulation, giving thus the impetus to grave organic changes.

Alcohol invariably reduces temperature ; an intoxicated man whose temperature is normal, or above normal, should be regarded as having some organic disease to account for it.

Referring to the after effects, he laid special stress on the alteration of tissues and accumulation of waste products, thus giving rise to various symptoms ascribed to, or diagnosed as neuralgia, rheumatism, rheumatic gout, spinal irritation, etc. If those cases of so-called rheumatic pains, which are relieved by the use of stimulants, were persuaded to drop them, and abstain entirely, their pains would, after the lapse of a few weeks' suffering, vanish entirely.

The principles of treating cases of alcoholism are, besides imperative self-control, first, restoration of digestive power—a difficult and tedious matter; then the restoration of the altered glands and tissues to their normal condition. The hallucinations and delusions which often accompany the disease are associated with capillary dilatation; and, as a sequence, there is pressure on nerve substance, with risk of subsequent atrophy or degeneration.

Those cases of fever which require alcohol in their treatment are marked by dry tongue and skin, and no indication of cerebro-spinal lesion. When a case is benefited by it, there is soon a lessened temperature, slower pulse, moister tongue and generally quieter condition.

Temporary administration may be resorted to when the surface has been chilled, or the powers of life weakened so that the heart is inadequate for its work. Its action then is like taking the pendulum off the clock—the spring is able to work so much faster.

The speaker concluded with the remark that alcohol was a good medicine, but a poor diet, and its action as a poison, visible in all ranks of society.

THE ACTION OF SCILLA.—Huseman. (*Deutsche Med. Wochens.—Practitioner*, May, 1878.)

While squill is one of the oldest known remedies, its mode of operation is still unknown. No alkaloid has yet been extracted from it, but it yields a substance resembling digitalin, which retards the action of the heart, without any inflammatory action upon stomach or intestines. Its emetic action it possesses in common with all cardiac poisons. In frogs it first induces slowing of the pulse, then arrest of the ventricle, arrest of the auricle and complete loss of irritability of the heart. The cord, nerves and muscles retain their excitability so that the animal can leap. In warm-blooded animals the double action characteristic of cardiac poisons is exhibited—upon the inhibitory nerves and upon the cardiac muscle itself. At the commencement of its action, it strengthens the heart's action, like all cardiac poisons (digitalis, etc.), and hence its diuretic action. It also at first increases arterial tension, and is therefore contra-indicated when this condition exists. The author never observed any trace of kidney inflammation, or anything which might suggest that squill was a direct stimulant to these organs. He was also unable to observe any indication of its possessing an expectorant action. In former times the dose was much larger than at present.

The Action of Potassium Salts.—Buchheim. (*Med. Chir. Rundschau.—Practitioner*, May, 1878.)

The action of the potassium salts differs much according to their diffusibility, the nitrate being readily diffusible, the sulphate and bicarbonate least so. With regard to the most easily diffusible salts, they overcome the arterial pressure of the capillaries of the stomach, causing the blood-corpuscles to accumulate in them, so that ecchymoses occur, or gastritis. These symptoms are less marked during digestion, or when the salts are in a state of dilution. The less diffusible salts pass into the small intestine, where they induce peristalsis, and are eliminated only after partial absorption. The sulphate forms the transition between the two groups. Potassium carbonate in large doses acts as a caustic on the mouth and stomach ; in the stomach is converted into a bicarbonate, and only acts as an aperient when the intestinal mucous membrane is very sensitive.

According to investigations, the ashes of blood serum contain only sodium salts, of blood corpuscles only potassium salts, while those of the albuminous tissues contain chiefly the latter ; so that the amount of potassic salts circulating in the blood is small, and all that is not combined must be quickly excreted by the kidneys. Hence the ingestion of considerable quantities must have marked effects—these are retardation of the heart-beats and final arrest in diastole. In frogs, muscular contractility is abolished, the excised heart stopping quickly in dilute potassium solutions—more slowly in those of sodium salts. The paralysis of the heart observed after injections of potassium salts is not a specific effect—as according to Podcopaew; but the direct action upon the first muscle with which they come in contact. Certain nerve-centers are similarly paralyzed with the muscles, in consequence of similar chemical composition. Death occurs, at times, however, before the occurrence of recognizable changes, because slight disturbance of these organs is enough to endanger life.

Indian Hemp in Migraine. (*L'Union Médicale du Canada, from Nice Médicale.*)

M. Seguin employs this remedy with success. The principle of this treatment consists in maintaining the nervous system under the slight influence of this medicine for a long time, as with the bromide in epilepsy. Two centigrams of the alcoholic extract are given to women before each meal. With men one may begin with three centigrams, finally four. In more than half of the cases the patients have been considerably benefited; some were completely cured. It may be said in conclusion, that Indian hemp has upon migraine the same influence that the bromide has upon epilepsy.

---

Dr. M. Mannheimer, of Chicago, has accomplished results in the treatment of whooping cough, which are of interest when considered in connection with an article on the same subject which we publish on another page. Dr. Mannheimer has employed intralaryngeal insufflations of a fine powder, composed of equal parts of the sulphate of quinine and white chalk. In nine cases the results were as follows:

| No. | Age. | Stage. | No. of Applications Daily. | Duration of Disease. | Remarks. |
|---|---|---|---|---|---|
| 1.. | 5 years... | Spasmodic | 1 | 5 days. | |
| 2.. | 5½ years. | Spasmodic | 1 | 6 days. | |
| 3.. | 7 years... | Spasmodic | 1 | 4 days. | |
| 4.. | 6 years... | Spasmodic | 2 | 7 days. | |
| 5.. | 12 years... | Catarrhal. | 2 | 7 days. | |
| 6.. | 16 years... | Spasmodic | 2 | 4 days .. | Syncope with each parox. |
| 7.. | 4¾ years. | Catarrhal. | 2 | 6 days. | |
| 8.. | 5¼ years. | Catarrhal. | 2 | 7 days. | |
| 9.. | 6¾ years. | Spasmodic | 2 | 8 days. | |
| | | | | 54 days. | |

It will be seen that the average duration of the disease, under this treatment, was six days.

